# Supplementary material for: MetaRibo-Seq measures translation in microbiomes
Source: Nat Commun. 2020 Jun 29;11:3268. doi: 10.1038/s41467-020-17081-z (PMC7324362; doi:10.1038/s41467-020-17081-z)
Supplement: Supplementary file 10 — Supplementary Data 7 [file 41467_2020_17081_MOESM10_ESM.zip › File2/Confidence_High_Taxonomy/377765_out.krona.html]

Javascript must be enabled to view this page.

members
magnitude
magnitudeUnassigned
count
unassigned
taxon
rank

377765\_out

16

2
superkingdom
16

1239
phylum
1

1
class
186801

186802
order
1

family
1
186803

1
genus
1432051

1720294

SRS146888\_contig\_number\_14497
species
1

phylum
1224

SRS075947\_contig\_number\_8747SRS098881\_contig\_number\_14038SRS143423\_contig\_number\_20881SRS146996\_contig\_number\_15833
15
4

11
class
1236

91347
11
order


SRS019129\_contig\_number\_5527SRS144219\_contig\_number\_contig-100\_787.788
543
family
2
11

9
1
genus

SRS140492\_contig\_number\_contig-100\_1966.160379
561

562

SRS012273\_contig\_number\_17276SRS017521\_contig\_number\_45729SRS049402\_contig\_number\_17164SRS064276\_contig\_number\_contig-100\_1091.183295SRS077646\_contig\_number\_contig-100\_469.470SRS142781\_contig\_number\_1901SRS144362\_contig\_number\_32267SRS147022\_contig\_number\_4558
species
8
